# Intramuscular (Infiltrating) Lipoma of the Floor of the Mouth

**DOI:** 10.1155/2018/3529208

**Published:** 2018-03-20

**Authors:** Ben-Zion Joshua, Lipa Bodner, Ruthy Shaco-Levy

**Affiliations:** ^1^Department of Otolaryngology Head and Neck Surgery, Soroka University Medical Center, Faculty of Health Sciences, Ben-Gurion University of the Negev, Beer-Sheva, Israel; ^2^Department of Oral and Maxillofacial Surgery, Faculty of Health Sciences, Ben-Gurion University of the Negev, Beer-Sheva, Israel; ^3^Institute of Pathology, Soroka University Medical Center, Faculty of Health Sciences, Ben-Gurion University of the Negev, Beer-Sheva, Israel

## Abstract

Lipoma is a very common soft tissue neoplasm, but only infrequently found in the oral region. Intramuscular lipoma (IML) is a relatively common variant of lipoma. The most common site for IML is the large muscles of the extremities, and it is quite rare in the oral cavity. A case of IML affecting the floor of the mouth/tongue of a 42-year-old female is described. The patient presented with a 4 cm diameter yellow mass in the right side of the sublingual area. Axial and coronal magnetic resonance imaging demonstrated its infiltrating nature that can be distinguished from the ordinary well-encapsulated lesion. The lesion was excised with adequate surgical margins. Histopathologically, the lesion was composed of mature adipose tissue that infiltrated the muscle in a diffuse manner. No lipoblasts, atypical cells, or high mitotic index were found. There was no evidence of recurrence two years postoperatively. Review of the literature yielded that IML occurring in the sublingual region is extremely rare.

## 1. Case

Lipomas are benign neoplasm composed of mature adipocytes. They are usually sharply circumscribed and possess some peripheral fibrous demarcation (pseudocapsule).

They are the most common tumor of the trunk and extremities. About 15–20% of cases involve the head and neck regions, while 1–4% affect the oral cavity [1, 2].

Oral lipomas (OLs) occur most commonly in the parotid region followed by the buccal mucosa, tongue, floor of the mouth, and palate. Histologically, lipomas are classified as classic lipoma, fibrolipoma, intramuscular lipoma (IML), angiolipoma, and spindle cell lipoma. OL usually presents as a slow-growing painless mass that rarely exceeds 20 mm in diameter [3]. The exact etiology of OL is unknown, although trauma and metaplasia of perivascular connective tissue have been suggested.

**Figure 1:**
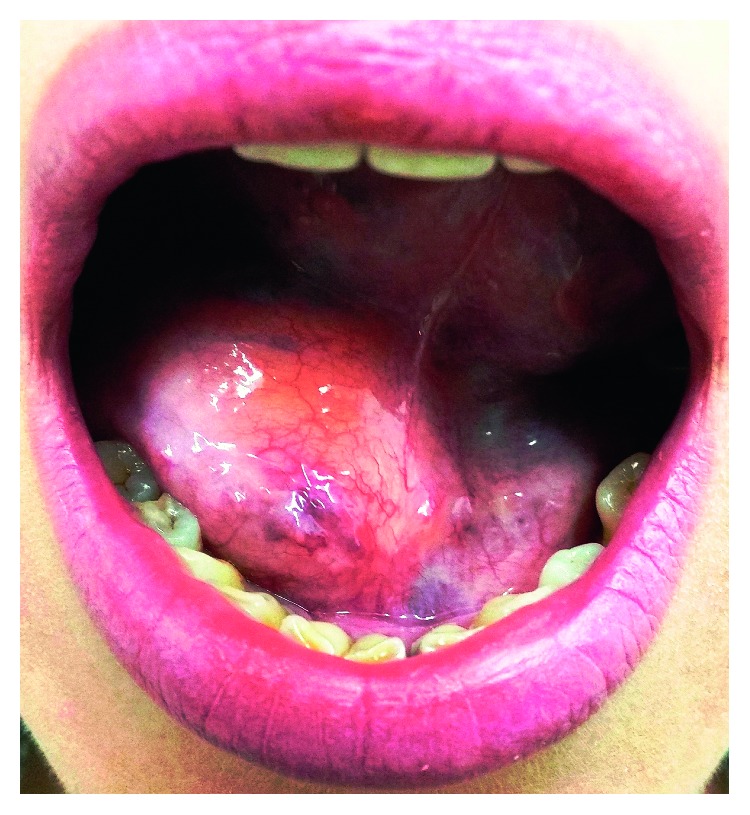
Preoperative image of the mouth reveals a huge soft tissue swelling affecting the right floor of the mouth.

**Figure 2:**
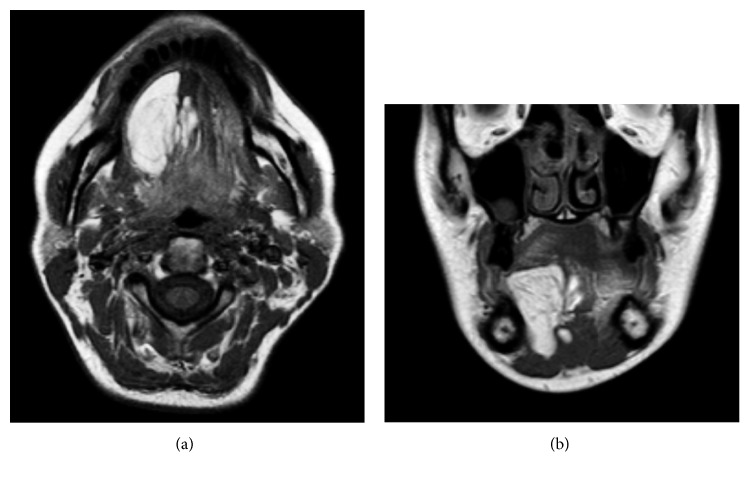
Axial (a) and coronal (b) MRI of the lesion (see text for description).

A 42-year-old female, generally healthy except for gastroesophageal reflux treated with lansoprazole 30 mg/day, presented with a 4.0 cm painless swelling of the right sublingual area that was growing slowly over three years. Clinical examination revealed a soft tissue mass, covered by the normal-appearing mucosa, with no ulceration (Figure 1). No cervical lymphadenopathy was noted. Axial and coronal magnetic resonance imaging (MRI) showed a 41 × 25 mm mass. The mass was hyperintense on T1 and underwent fat suppression. It involved the right floor of the mouth and tongue including the genioglossus, the hyoglossus, the mylohyoid, and the intrinsic muscles of the tongue (Figure 2). The clinical and radiological findings were consistent with a lipoma, but a liposarcoma was part of the differential diagnosis. The patient was advised to undergo a complete excision to which he consented.

Under general anesthesia, via an incision in the right floor of the mouth, the tumor was removed, together with a thin margin of normal muscle tissue, in the inferior aspect and some normal oral mucosa in the superior aspect.

The surgical specimen consisted of a soft yellow-white mass with smooth external surfaces and a uniform solid yellow cut surface.

**Figure 3:**
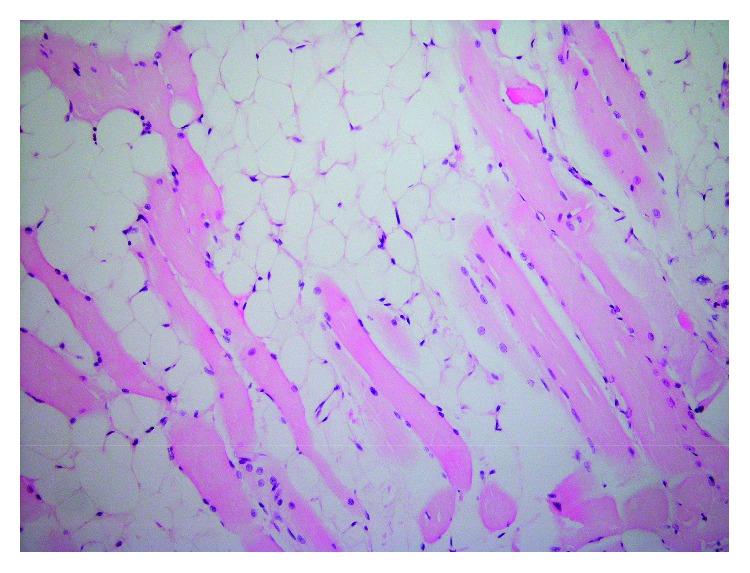
Histopathology ofmature adipose tissue with striated muscle fibers interspersed within (hematoxylin and eosin, original magnification ×200).

Microscopic examination revealed mature adipose tissue with striated muscle fibers interspersed within (Figure 3). There was no evidence of cytological atypia, increased cellularity, or necrosis. In addition, all sections consisted of mature adipose tissue; no lipoblasts were identified. The final diagnosis was intramuscular (infiltrating) lipoma.

**Figure 4:**
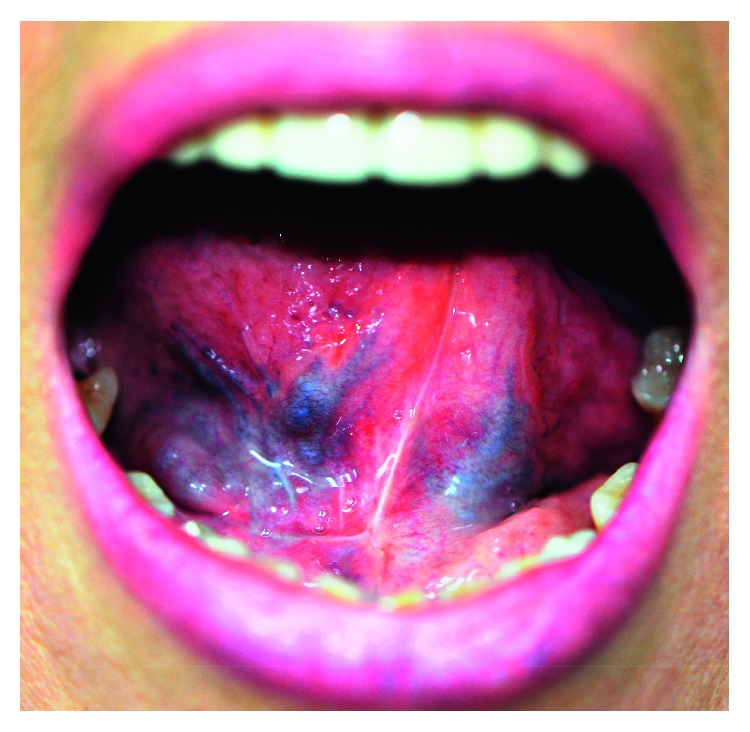
Postoperative image of the mouth reveals a normal appearance of the tongue and floor of the mouth.

The postoperative course was uneventful. Two years after surgery, the patient has no evidence of recurrence (Figure 4).

## 2. Discussion

Oral lipomas (OLs) commonly present as a soft, mobile, lobulated, yellow mass. It is usually observed among adults, between the age of 40 and 60 years, with no gender preference. The most common site for OL is the buccal mucosa, a region that is normally rich with adipose tissue, followed by the tongue, lips, floor of the mouth, palate, and gingiva [3].

OL consists of mature adipocytes organized into lobules that are separated by septa of fibrous tissue. Although the OL is morphologically indistinguishable from normal fat, it differs from normal body fat, in that lipids contained in the OL are metabolically inactive [4].

Oral IML seems to be larger in size and displays an infiltrative pattern of growth, as compared with the classic OL. It is characterized by a much deeper localization, slow-growing, painless mass that often can cause swelling and deformity. Its consistency is often rubbery. In the rare occasion, when the infiltration is very extensive, it may cause local muscle dysfunction or sensory disturbance, due to local pressure on a branch of the trigeminal nerve [5].

Oral IMLs are very rare and are described most commonly in the tongue [6, 7, 8].

Local recurrence rate for the IML is reported as high as 63% [6, 9]. This high rate is probably related to the infiltrative nature of the tumor.

Due to the infiltrative-like pattern of growth of IML, it may be difficult to distinguish IML from a well-differentiated liposarcoma, just by means of diagnostic imaging modalities (ultrasound (US), computerized tomography (CT), or MRI). Hence, the only way to obtain a diagnosis is by an incisional or excisional biopsy. In this case, the mass was completely resected without a biopsy. The authors felt that the lesion could be completely excised. In spite of the tumor appearing to invade the muscles, the degree of invasion based on palpation was limited, and the authors felt that the lesion involved some muscle fibers that could be completely excised without compromising the overall structure or function of the involved muscles. Alternatively obtaining an incisional or trucut biopsy before complete resection could be considered reasonable as well.

The mass of IML may present high-signal intensity with some strands of low-signal intensity, which may refer to the remnants of the lingual muscle fibers. CT appearance of a well-differentiated liposarcoma may be very similar to the remnants of infiltrated soft tissue [10]. Despite the growth pattern similarity between IML and well-differentiated liposarcoma/atypical lipomatous tumor, it can usually be differentiated. Adipocytes contain a large lipid droplet surrounded by a layer of cytoplasm. The nucleus is flattened and located on the periphery, whereas lipoblasts characteristically have abundant multivacuolated clear cytoplasm and a dark staining (hyperchromatic), indented nucleus. Lipoma rather than liposarcoma is diagnosed If the lesion does not show any of the following: lipoblastic proliferation, variation in adipocyte size, atypical and enlarged adipocyte nuclei, hyperchromatic, or bizarre stromal cells in fibrous septa, between adipocytes or in vessel walls [11, 12].

One more infiltrative lipoma to consider is the spindle cell lipoma which may occur occasionally in the tongue. Although it rarely infiltrates adjacent tissue, there are variants with predominance of the adipocyte component and unusual forms of multifocal disease [13] which may seem similar microscopically to IML.

## 3. Conclusion

Oral lipoma is quite rare. The variant of intramuscular (infiltrating) lipoma should be kept in mind and should be differentiated microscopically from liposarcoma. Complete surgical excision with minimal morbidity is recommended.
